# (*E*)-*O*-Isopropyl *N*-(4-nitro­phen­yl)thio­carbamate

**DOI:** 10.1107/S1600536807067360

**Published:** 2007-12-21

**Authors:** Carol A. Ellis, Edward R. T. Tiekink, Julio Zukerman-Schpector

**Affiliations:** a Department of Chemistry, The University of Texas at San Antonio, One UTSA Circle, San Antonio, Texas 78249-0698, USA; b Department of Chemistry, Universidade Federal de São Carlos, 13565-905 São Carlos, SP, Brazil

## Abstract

The configuration of the thione–aryl C—N single bond in the title mol­ecule, C_10_H_12_N_2_O_3_S, is *E*. Centrosymmetrically related mol­ecules are connected into a dimer *via* an eight-membered thio­amide {⋯H—N—C=S}_2_ synthon and mol­ecules are consolidated into the crystal structure *via* C—H⋯O inter­actions.

## Related literature

For related structures, see: Ho *et al.* (2005 ▶); Kuan *et al.* (2007 ▶). For related literature, see: Ho *et al.* (2006 ▶); Ho & Tiekink (2007 ▶).
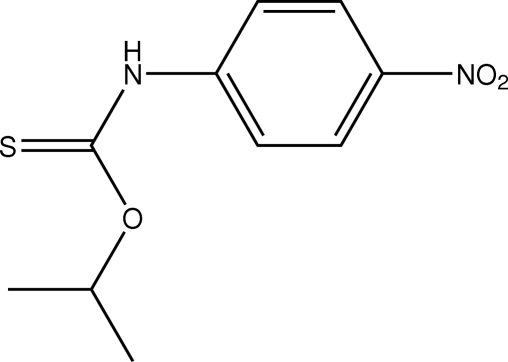


## Experimental

### 

#### Crystal data

C_10_H_12_N_2_O_3_S
*M* *_r_* = 240.28
Triclinic, 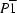

*a* = 7.4200 (8) Å
*b* = 8.3206 (9) Å
*c* = 10.0993 (11) Å
α = 111.414 (4)°
β = 97.877 (5)°
γ = 94.130 (5)°
*V* = 569.94 (11) Å^3^
*Z* = 2
Mo *K*α radiation
μ = 0.28 mm^−1^
*T* = 98 (2) K
0.30 × 0.18 × 0.10 mm

#### Data collection

Rigaku AFC12κ/SATURN724 diffractometer
Absorption correction: multi-scan (*ABSCOR*; Higashi, 1995 ▶) *T*_min_ = 0.915, *T*_max_ = 1 (expected range = 0.890–0.973)
3875 measured reflections
2221 independent reflections
2103 reflections with *I* > 2σ(*I*)
*R*_int_ = 0.024

#### Refinement

*R*[*F* ^2^ > 2σ(*F* ^2^)] = 0.066
*wR*(*F* ^2^) = 0.119
*S* = 1.33
2221 reflections
145 parameters
H-atom parameters constrained
Δρ_max_ = 0.29 e Å^−3^
Δρ_min_ = −0.24 e Å^−3^

### 

Data collection: *CrystalClear* (Rigaku, 2005 ▶); cell refinement: *TwinSolve* (Rigaku and Prekat AB, 2006 ▶); data reduction: *TwinSolve*; program(s) used to solve structure: *SHELXS97* (Sheldrick, 1997 ▶); program(s) used to refine structure: *SHELXL97* (Sheldrick, 1997 ▶); molecular graphics: *ORTEPII* (Johnson, 1976 ▶) and *DIAMOND* (Brandenburg, 2006 ▶); software used to prepare material for publication: *SHELXL97*.

## Supplementary Material

Crystal structure: contains datablocks global, I. DOI: 10.1107/S1600536807067360/ng2410sup1.cif

Structure factors: contains datablocks I. DOI: 10.1107/S1600536807067360/ng2410Isup2.hkl

Additional supplementary materials:  crystallographic information; 3D view; checkCIF report

## Figures and Tables

**Table 1:** Hydrogen-bond geometry (Å, °)

*D*—H⋯*A*	*D*—H	H⋯*A*	*D*⋯*A*	*D*—H⋯*A*
N1—H1n⋯S1^i^	0.88	2.59	3.440 (2)	162
C7—H7⋯O2^ii^	0.95	2.48	3.204 (4)	133
C8—H8⋯O3^iii^	1.00	2.50	3.275 (4)	134

Symmetry codes: (i) 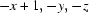; (ii) 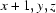; (iii) 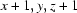.

**Fig. 1.**
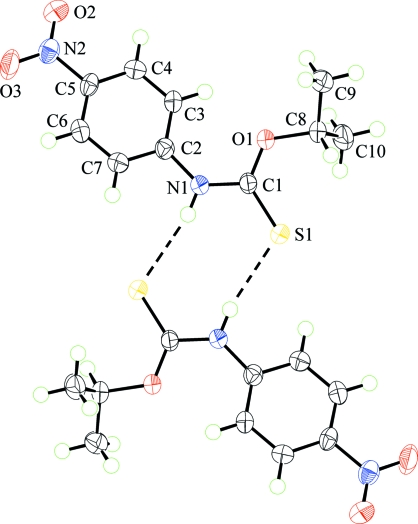
Dimer formation in (I) mediated by N—H···S hydrogen bonding (dashed lines) between the centrosymmetrically related molecules, showing atom labelling and displacement ellipsoids at the 70% probability level. The unlabelled atoms are related to the labelled atoms by 1 - x, -y, -z.

**Fig. 2.**
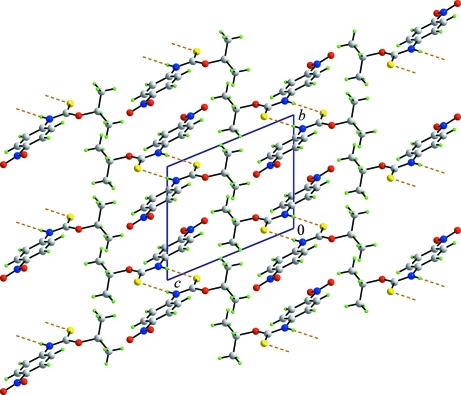
Crystal packing in (I) viewed down the a axis. N—H···S hydrogen bonding is shown as orange dashed lines.

## supplementary crystallographic information

### Comment

The title thiocarbamate (I), Fig. 1, was investigated as a part of an on-going evaluation of the structural features of these molecules (Ho *et al.*, 2005; Kuan *et al.*, 2007) along with their phosphinegold(I) complexes (Ho *et al.*, 2006; Ho & Tiekink, 2007). While the central portion of the molecule is planar, small twists are evident in the outer extremities as seen in the N1/C1/O1/H8 and C1/N1/C2/C3 torsion angles of 33 and -20.0 (5)°, respectively. Geometric parameters resemble those found in related systems (Ho *et al.*, 2005; Kuan *et al.*, 2007). The key synthon in the crystal structure is the eight-membered thioamide {···H—N—C=S}_2_ synthon, Table 1, as found in most thiocarbamate structures (Kuan *et al.*, 2007). The dimers thus formed stack into layers along the *c* axis with the alkyl groups projecting on either side to from aliphatic layers. Interactions of the type C—H···O, involving each of the nitro-O atoms, are formed within and between layers, Table 1.

### Experimental

Compound (I) was prepared by refluxing 4-nitrophenylisothiocyanate (Aldrich) with 2-propanol using standard methods (Ho *et al.*, 2005). The yellow precipitate, which was obtained upon concentration of the reaction solution, was dissolved in diethyl ether and layered with hexane in a 1:1 ratio. Yellow crystals were isolated by slow evaporation; m. p. 465 - 469 K. IR (cm^-1^): ν(N—H) 3241 (br), ν(NO_2_) 1549 (*s*), ν(CN) 1494 (*s*), ν(NO_2_) (*s*), ν(CS) 1083 (*s*). NMR (DMSO, p.p.m.): δ 1.37 (6 H, d, J = 6.2 Hz, CH_3_), 5.56 (1 H, septet, J = 6.2 Hz, CH), 7.78 (2 H, br, aryl-H), 8.21 (2 H, br, aryl-H), 11.52 (1 H, br, NH).

### Refinement

All H atoms were included in the riding-model approximation with C—H = 0.95 to 1.00 Å and N—H = 0.88 Å, and with *U*_iso_(H) = 1.5*U*_eq_(methyl-C) or 1.2*U*_eq_(N and remaining-C).

### Crystal data

**Table:**

C_10_H_12_N_2_O_3_S	*Z* = 2
*M**_r_* = 240.28	*F*_000_ = 252
Triclinic, *P*1	*D*_x_ = 1.400 Mg m^−^^3^
Hall symbol: -P 1	Mo *K*α radiation λ = 0.71070 Å
*a* = 7.4200 (8) Å	Cell parameters from 4435 reflections
*b* = 8.3206 (9) Å	θ = 2.6–29.2º
*c* = 10.0993 (11) Å	µ = 0.28 mm^−^^1^
α = 111.414 (4)º	*T* = 98 (2) K
β = 97.877 (5)º	Block, yellow
γ = 94.130 (5)º	0.30 × 0.18 × 0.10 mm
*V* = 569.94 (11) Å^3^	

### Data collection

**Table:**

Rigaku AFC12κ/SATURN724 diffractometer	2221 independent reflections
Radiation source: fine-focus sealed tube	2103 reflections with *I* > 2σ(*I*)
Monochromator: graphite	*R*_int_ = 0.024
*T* = 98(2) K	θ_max_ = 26.0º
ω scans	θ_min_ = 2.7º
Absorption correction: multi-scan(ABSCOR; Higashi, 1995)	*h* = −9→9
*T*_min_ = 0.915, *T*_max_ = 1	*k* = −10→10
3875 measured reflections	*l* = −12→12

### Refinement

**Table:**

Refinement on *F*^2^	Secondary atom site location: difference Fourier map
Least-squares matrix: full	Hydrogen site location: inferred from neighbouring sites
*R*[*F*^2^ > 2σ(*F*^2^)] = 0.066	H-atom parameters constrained
*wR*(*F*^2^) = 0.119	*w* = 1/[σ^2^(*F*_o_^2^) + (0.0191*P*)^2^ + 0.7747*P*] where *P* = (*F*_o_^2^ + 2*F*_c_^2^)/3
*S* = 1.33	(Δ/σ)_max_ < 0.001
2221 reflections	Δρ_max_ = 0.29 e Å^−^^3^
145 parameters	Δρ_min_ = −0.24 e Å^−^^3^
Primary atom site location: structure-invariant direct methods	Extinction correction: none

### Special details

**Table:**

Geometry. All e.s.d.'s (except the e.s.d. in the dihedral angle between two l.s. planes) are estimated using the full covariance matrix. The cell e.s.d.'s are taken into account individually in the estimation of e.s.d.'s in distances, angles and torsion angles; correlations between e.s.d.'s in cell parameters are only used when they are defined by crystal symmetry. An approximate (isotropic) treatment of cell e.s.d.'s is used for estimating e.s.d.'s involving l.s. planes.
Refinement. Refinement of *F*^2^ against ALL reflections. The weighted *R*-factor *wR* and goodness of fit *S* are based on *F*^2^, conventional *R*-factors *R* are based on *F*, with *F* set to zero for negative *F*^2^. The threshold expression of *F*^2^ > σ(*F*^2^) is used only for calculating *R*-factors(gt) *etc*. and is not relevant to the choice of reflections for refinement. *R*-factors based on *F*^2^ are statistically about twice as large as those based on *F*, and *R*- factors based on ALL data will be even larger.

### Fractional atomic coordinates and isotropic or equivalent isotropic displacement parameters (Å^2^)

**Table:**

	*x*	*y*	*z*	*U*_iso_*/*U*_eq_	
S1	0.55527 (11)	0.09264 (11)	0.23408 (8)	0.0271 (2)	
O1	0.2493 (3)	0.2303 (3)	0.3002 (2)	0.0227 (4)	
O2	−0.4727 (3)	0.3762 (3)	−0.1315 (2)	0.0369 (6)	
O3	−0.3181 (3)	0.3892 (3)	−0.2947 (2)	0.0391 (6)	
N1	0.2838 (3)	0.1543 (3)	0.0701 (2)	0.0238 (5)	
H1N	0.3503	0.1049	0.0042	0.029*	
N2	−0.3330 (3)	0.3620 (3)	−0.1846 (3)	0.0268 (6)	
C1	0.3532 (4)	0.1629 (4)	0.2037 (3)	0.0215 (6)	
C2	0.1242 (4)	0.2101 (4)	0.0165 (3)	0.0216 (6)	
C3	−0.0289 (4)	0.2438 (4)	0.0835 (3)	0.0247 (6)	
H3	−0.0301	0.2328	0.1737	0.030*	
C4	−0.1797 (4)	0.2937 (4)	0.0174 (3)	0.0241 (6)	
H4	−0.2850	0.3168	0.0618	0.029*	
C5	−0.1743 (4)	0.3091 (4)	−0.1142 (3)	0.0218 (6)	
C6	−0.0249 (4)	0.2768 (4)	−0.1820 (3)	0.0255 (6)	
H6	−0.0240	0.2889	−0.2719	0.031*	
C7	0.1240 (4)	0.2261 (4)	−0.1162 (3)	0.0256 (6)	
H7	0.2279	0.2018	−0.1621	0.031*	
C8	0.3146 (4)	0.2614 (4)	0.4537 (3)	0.0244 (6)	
H8	0.4509	0.2935	0.4777	0.029*	
C9	0.2226 (5)	0.4141 (4)	0.5385 (3)	0.0306 (7)	
H9A	0.2637	0.5168	0.5193	0.046*	
H9B	0.2557	0.4388	0.6419	0.046*	
H9C	0.0891	0.3852	0.5088	0.046*	
C10	0.2626 (5)	0.0986 (4)	0.4801 (3)	0.0331 (7)	
H10A	0.3280	0.0054	0.4248	0.050*	
H10B	0.1300	0.0627	0.4494	0.050*	
H10C	0.2962	0.1218	0.5833	0.050*	

### Atomic displacement parameters (Å^2^)

**Table:**

	*U*^11^	*U*^22^	*U*^33^	*U*^12^	*U*^13^	*U*^23^
S1	0.0270 (4)	0.0355 (4)	0.0209 (4)	0.0135 (3)	0.0040 (3)	0.0115 (3)
O1	0.0240 (10)	0.0282 (11)	0.0172 (10)	0.0079 (8)	0.0036 (8)	0.0095 (8)
O2	0.0287 (12)	0.0482 (15)	0.0355 (13)	0.0145 (11)	0.0051 (10)	0.0161 (11)
O3	0.0336 (13)	0.0576 (16)	0.0340 (13)	0.0068 (11)	−0.0020 (10)	0.0294 (12)
N1	0.0260 (13)	0.0285 (13)	0.0176 (12)	0.0101 (10)	0.0044 (10)	0.0083 (10)
N2	0.0272 (14)	0.0238 (13)	0.0262 (13)	0.0006 (10)	−0.0026 (11)	0.0089 (11)
C1	0.0269 (15)	0.0216 (14)	0.0161 (13)	0.0057 (12)	0.0023 (11)	0.0072 (11)
C2	0.0229 (14)	0.0199 (14)	0.0184 (14)	0.0013 (11)	−0.0002 (11)	0.0049 (11)
C3	0.0262 (15)	0.0313 (16)	0.0191 (14)	0.0066 (12)	0.0029 (11)	0.0125 (12)
C4	0.0235 (15)	0.0253 (15)	0.0238 (15)	0.0058 (12)	0.0072 (12)	0.0082 (12)
C5	0.0233 (14)	0.0183 (14)	0.0209 (14)	0.0007 (11)	−0.0039 (11)	0.0071 (11)
C6	0.0303 (16)	0.0275 (16)	0.0190 (14)	0.0029 (12)	0.0032 (12)	0.0097 (12)
C7	0.0234 (15)	0.0310 (16)	0.0228 (15)	0.0062 (12)	0.0056 (12)	0.0098 (13)
C8	0.0256 (15)	0.0311 (16)	0.0175 (14)	0.0065 (12)	0.0014 (11)	0.0109 (12)
C9	0.0358 (17)	0.0305 (17)	0.0258 (16)	0.0099 (14)	0.0080 (13)	0.0093 (13)
C10	0.0429 (19)	0.0363 (18)	0.0262 (16)	0.0100 (15)	0.0087 (14)	0.0171 (14)

### Geometric parameters (Å, °)

**Table:**

S1—C1	1.678 (3)	C4—H4	0.9500
O1—C1	1.314 (3)	C5—C6	1.373 (4)
O1—C8	1.479 (3)	C6—C7	1.383 (4)
O2—N2	1.224 (3)	C6—H6	0.9500
O3—N2	1.231 (3)	C7—H7	0.9500
N1—C1	1.352 (3)	C8—C10	1.508 (4)
N1—C2	1.413 (4)	C8—C9	1.517 (4)
N1—H1N	0.8800	C8—H8	1.0000
N2—C5	1.470 (4)	C9—H9A	0.9800
C2—C7	1.394 (4)	C9—H9B	0.9800
C2—C3	1.395 (4)	C9—H9C	0.9800
C3—C4	1.391 (4)	C10—H10A	0.9800
C3—H3	0.9500	C10—H10B	0.9800
C4—C5	1.387 (4)	C10—H10C	0.9800
			
C1—O1—C8	119.8 (2)	C5—C6—H6	120.9
C1—N1—C2	131.9 (2)	C7—C6—H6	120.9
C1—N1—H1N	114.0	C6—C7—C2	121.1 (3)
C2—N1—H1N	114.0	C6—C7—H7	119.4
O2—N2—O3	123.5 (3)	C2—C7—H7	119.4
O2—N2—C5	118.7 (2)	O1—C8—C10	109.3 (2)
O3—N2—C5	117.8 (2)	O1—C8—C9	105.0 (2)
O1—C1—N1	113.7 (2)	C10—C8—C9	112.9 (3)
O1—C1—S1	126.2 (2)	O1—C8—H8	109.8
N1—C1—S1	120.2 (2)	C10—C8—H8	109.8
C7—C2—C3	119.6 (3)	C9—C8—H8	109.8
C7—C2—N1	115.1 (3)	C8—C9—H9A	109.5
C3—C2—N1	125.2 (3)	C8—C9—H9B	109.5
C4—C3—C2	119.6 (3)	H9A—C9—H9B	109.5
C4—C3—H3	120.2	C8—C9—H9C	109.5
C2—C3—H3	120.2	H9A—C9—H9C	109.5
C5—C4—C3	119.0 (3)	H9B—C9—H9C	109.5
C5—C4—H4	120.5	C8—C10—H10A	109.5
C3—C4—H4	120.5	C8—C10—H10B	109.5
C6—C5—C4	122.4 (3)	H10A—C10—H10B	109.5
C6—C5—N2	118.3 (3)	C8—C10—H10C	109.5
C4—C5—N2	119.4 (3)	H10A—C10—H10C	109.5
C5—C6—C7	118.2 (3)	H10B—C10—H10C	109.5
			
C8—O1—C1—N1	−175.0 (2)	O2—N2—C5—C6	173.7 (3)
C8—O1—C1—S1	4.4 (4)	O3—N2—C5—C6	−6.1 (4)
C2—N1—C1—O1	2.6 (4)	O2—N2—C5—C4	−6.4 (4)
C2—N1—C1—S1	−176.8 (3)	O3—N2—C5—C4	173.8 (3)
C1—N1—C2—C7	162.0 (3)	C4—C5—C6—C7	0.4 (4)
C1—N1—C2—C3	−19.9 (5)	N2—C5—C6—C7	−179.7 (3)
C7—C2—C3—C4	−0.2 (4)	C5—C6—C7—C2	−0.8 (4)
N1—C2—C3—C4	−178.3 (3)	C3—C2—C7—C6	0.7 (4)
C2—C3—C4—C5	−0.1 (4)	N1—C2—C7—C6	178.9 (3)
C3—C4—C5—C6	0.0 (4)	C1—O1—C8—C10	−87.1 (3)
C3—C4—C5—N2	−179.9 (3)	C1—O1—C8—C9	151.5 (3)

### Hydrogen-bond geometry (Å, °)

**Table:**

*D*—H···*A*	*D*—H	H···*A*	*D*···*A*	*D*—H···*A*
N1—H1n···S1^i^	0.88	2.59	3.440 (2)	162
C7—H7···O2^ii^	0.95	2.48	3.204 (4)	133
C8—H8···O3^iii^	1.00	2.50	3.275 (4)	134

Symmetry codes: (i) −*x*+1, −*y*, −*z*; (ii) *x*+1, *y*, *z*; (iii) *x*+1, *y*, *z*+1.
